# Health Risk Assessment of Metals (Cu, Pb, Zn, Cr, Cd, As, Hg, Se) in Angling Fish with Different Lengths Collected from Liuzhou, China

**DOI:** 10.3390/ijerph17072192

**Published:** 2020-03-25

**Authors:** Jun Li, Xiongyi Miao, Yupei Hao, Zhouqing Xie, Shengzheng Zou, Changsong Zhou

**Affiliations:** 1 Key Laboratory of Karst Dynamics, MNR&GZAR, Institute of Karst Geology, CAGS, Guilin 541004, China; lipsgeology@karst.ac.cn (J.L.); zshzh@karst.ac.cn (S.Z.); changsongzhou@karst.ac.cn (C.Z.); 2 Department of Municipal and Environmental Engineering, Hebei University of Architecture, Zhangjiakou 075000, China; 3 Institute of Polar Environment, School of Earth and Space Sciences, University of Science and Technology of China, Hefei 230026, China; zq_xie18@163.com

**Keywords:** fish caught by anglers, metals, different lengths, health risk assessments, Liuzhou

## Abstract

Wild fish caught by anglers (WFAs) were confirmed to be usually contaminated with metals, and the contamination status is radically affected by the growth and length of the fish. To determine the contamination levels of metals and health risks in WFAs with different length ranges of fish, this study ascertained the concentration of eight metals, including Cu, Pb, Zn, Cr, Cd, As, Hg and Se, in 171 wild fishes collected from the watershed of Liujiang River. The assessment of metal pollution and health risks from the consumption of these fishes with seven length ranges were accomplished. The obtained results implied a relatively high concentration of Zn, Cr, and Cd up to 109.294 mg/kg, 4.226 mg/kg, and 0.196 mg/kg (wet weight), respectively, which exceed the corresponding Maximum Residue Limit (MRL). The negative correlation between Cu, Zn, Cr, and Cd was observed to be significant with fish length, signifying a possible occurrence of biological dilution on these metals. The WFAs were mostly contaminated with Cr and Cd irrespective of the length ranges of fish, which were denoted by the average pollution index (Pi) of Cr and Cd and were commonly found to be beyond 0.2. Based on the results of health risk assessment analysis, most of the target hazard quotient (THQ) values of Cr were below 1, implying that the consumption of wild fish for adults has insignificant health risk. For children, the total target hazard quotient (TTHQ) values of beyond 1 were found in fishes with the length range of <25 cm, particularly a TTHQ value 1.627 in the range of 10–15 cm, indicating that children are being prone to serious health risks owing to the consumption of WFAs. The weekly recommended consumption of WFAs with the length range of 10–15 cm for adults and children was 0.298 kg/week and 0.149 kg/week, respectively. These are substantially lower than the current rate of fish consumption (0.42 kg/week), and therefore, the wild fish with the length range of 10–15 cm should be avoided for consumption.

## 1. Introduction

Fish and their products are rich in proteins, microelements, omega-3 fatty acids, and low saturated fat, which are easily digested and absorbed by humans [1,2]. Hence, they are popularly consumed by people worldwide and one of the primary sources of nutrients [3]. However, the abuse of antibiotics, hormones, and illicit drugs in fish cultivation raises concerns on the consumption of cultivated fish [4,5]. Given that the wild fish are fed naturally and grown slowly, wild fish are perceived to be healthier for human nutrition, and hence their consumption is highly sought. The wild fish caught from the remote waterways are healthier and safe to consume as they are less polluted by metals. However, in aquatic ecosystems, wild fish are present at the top of the food chain and are susceptible to metals pollution. Previous investigations have confirmed that all over the world wild fish commonly have one or several metals beyond the safety thresholds (i.e. Cr, Cd, Zn) [6,7,8,9], especially in the developing countries. The consumption of wild fish contaminated with metals not only offset the benefits of eating fish, but also cause metals poisoning. Therefore, investigations on the wild fish could be beneficial to restrain the exaggerated and misled advertisements about the consumption of wild fish.

In most of the large cities, angling is not only an ideal recreational activity but also a vital way to obtain wild fish [9]. There are about 90 million fishing enthusiasts in China, as indicated by the Chinese Fishing Association, and many anglers can often be found scattered along the banks of rivers in the cities and suburbs. Given the scarcity of wild fish, the wild fish caught by anglers (WFAs) are mainly for personal consumption or sale to others, only on a small-scale. However, the health risks due to the consumption of WFAs may not be as high as it seems to be. Previous studies indicated that living and preying habitats of wild fish easily make them enriched with various metals, but such enrichment is largely different between the species, growth, living and feeding behaviors, etc. [10,11,12]. Even though the impacts of the species and living and feeding behaviors of WFAs on the enrichment of metals were reported earlier, the studies focusing on the estimation of different health risks among WFAs through growth are still lacking, and hence qualitatively identifying the health risks resulting from the consumption of WFAs is critical. The significant relationship between fish length as an essential indicator of fish growth and concentration of metal has been reported [13,14], which is strongly related to the metabolism of metals and dilution of fat. The juvenile fish of small size usually have a higher concentration of metals, which could be attributed to the low metabolic rate of metals arising from the underdeveloped organs. On the contrary, the concentration of metals in adult fish with a larger size is often low, which could be due to the intense metabolic capability to excrete excess metals from the body and higher accumulation of fat. Although, due to this reason, it is believed that adult fish with a larger size is safer than juvenile fish with a smaller size for human consumption, the detailed health risks of WFAs in different lengths of fish are still uncertain. Therefore, with the regulation of angling activities is missing, it is essential to determine the ranges of fish length that have higher health risks for human consumption.

Liuzhou is the largest industrial city in Guangxi province and has an integrated industrial system and important economic status. However, the annual emission of sewage from this city is more than 350 million tons, most of which come from the emission of metal smelting, food, chemical, and paper industries [9]. The dispersed industrial sewage finally converges into Liujiang River and its tributary of Luoqingjiang River, which are the major surface waterways in this city. Hence, metals are the key contaminants in Liujiang River and its tributary Luoqingjiang River, especially in Liujiang River [9,15,16]. However, the pollution of metals in these rivers has not deterred anglers from fishing, and many residents are still angling fish from these rivers. Despite the contamination of metals in angling fish that have already been confirmed in our previous study, it is still unclear about the variation in the contamination of metals in WFAs with different sizes. Also, only limited information is available about the safer length ranges of wild fish for anglers and consumers. For this purpose, the study aimed to investigate of eight metals (Cu, Pb, Zn, Cr, Cd, As, Hg and Se) in WFAs with different length ranges, and the evaluation of WFAs with different length ranges that could be useful to determine the suitable and acceptable length of fish to angle further. The study also aimed to use the current information as means for environmental monitoring and the effect of pollution.

## 2. Materials and Methods

### 2.1. Study Sites and Fish Sampling

Liujiang River and its tributary Luoqingjiang River are the most important surface watersheds in Liuzhou city. Liujiang River is also the largest river in Liuzhou city and the second-largest tributary of the Xijiang River in the Pearl River Basin [9]. These rivers cover the urban and suburb areas of Liuzhou. However, mining industries are widely distributed in the upper reaches of the Liujiang River, and the extensive mining activities have increased the concentration of metals in these rivers [17]. The middle reaches of Liujiang River flow through most of the functional areas of Liuzhou city, which include main industrial areas, city center, and residential areas. Hence, the metals pollution of rivers distributed in the Liuzhou city is stemmed from industrial wastewater and domestic sewage. Although Longqingjiang River flows mainly through agricultural zones without large-scale industries, some industrial parts and partially industrial areas in the east suburban of Liuzhou city are also found in the watershed areas of Luoqingjiang River [9]. Hence, it can be speculated that the watersheds of the Liujiang River may be in general, suffer from metals pollution to some extent.

**Figure 1:**
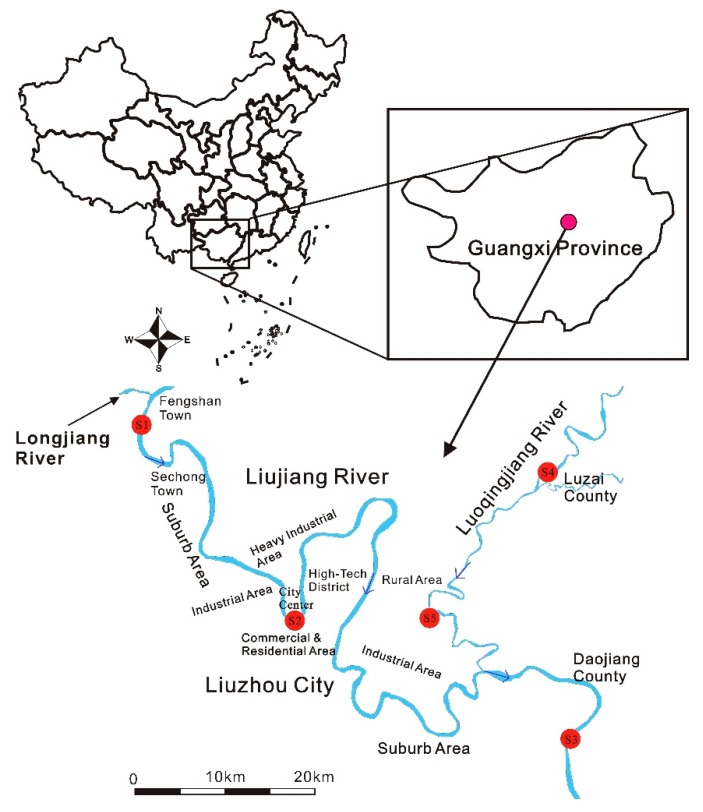
Study area with the five sampling sites along the Liujiang River and its tributary the Luoqingjiang River.

In order to reveal the contamination of wild fish more rigorously, in this study area, five angling areas were selected along the banks of Liujiang River and its tributary Luoqingjiang River. The sampling points are shown in Figure 1 (modified from [9]). Three points (S1, S2, and S3) were used to control the upstream (Fengshan Town), midstream (Liuzhou city), and downstream (DaoJiang county) of Liujiang River, whereas, the other two (S4 and S5) of them were located in Luzhai county and Sanchawan, respectively. The fish samples were directly obtained from the anglers located in every selected angling area, from 12–18 May 2019. The fishing ban is from 1 April to 1 June every year, as indicated by the people’ government of Liuzhou city. During the sampling period, commercial fishing was banned, but only individual angling activities were allowed. Hence, there were many anglers in the angling areas on both sides of the Lujiang and Luoqingjiag Rivers, which facilitated the collection of samples. All fish samples were collected directly from different anglers in every sampling site. Also, fish samples with reasonable quantity, species, and size were collected as far as possible. The information about fish length, location, and species were recorded during the collection of fish samples, followed by storage of them in a self-sealed polythene bag under −20 ℃. A total of 171 fish samples were quickly transferred to the laboratory for the determination of 8 metals in WFAs. All fish samples, classified into 14 species, were obtained from the study areas of S1–S5. The obtained WFAs with the length range of 10–20 cm that were distributed at all the sampling points accounted for 49.1% of the total fish samples. The details of the fish samples are shown in Table 1. All fish samples were divided into four groups of *Pseudohemiculter dispar*, *Cyprinus carpio*, *Ervthroculter hypselonotus,* and others (remaining fish species), and the length ranges of which were distributed from 5–37 cm, 8–41 cm, 6–34 cm, and 8.5–51 cm, respectively. The length ranges of the fish samples in each group were relatively similar, which is beneficial to reveal the concentration of metals in the fish bodies with different lengths.

### 2.2. Sample Preparation and Analysis

The body weight and length of WFAs were measured, and the species of WFAs were identified, and an autopsy was conducted in the laboratory. Then, 50–100 g of muscle tissues were extracted from the back of the fish samples. In order to meet the minimal detection limit, the muscle tissues of more than three small samples, which were maintained at the same sampling site and specie, with approximate length or weight, were mixed. The muscle tissues were then washed by Milli-Q water and further freeze dried at −80 ℃ for 72 h to obtain constant weight. A grinder was used to powder the dried muscle tissue for uniform particle size and then frozen at −20 ℃ until subsequent analysis. The details of the above procedure were described by Fu et al. [18]. Inductively coupled plasma mass spectrometry (ICP-MS, Thermo X series, ThermoFisher, Waltham, MA, USA) method was used to measure the concentrations of Cd, Cr, Cu, Pb, Zn, and Se, while the concentrations of Hg and As were determined by an atomic fluorescence spectrometry method (AFS-920, Titan instruments, Shanghai, China). In order to maintain the quality assurance and quality control, the certified reference material (GBW10018), reagent blank, and duplicates of the prepared samples were used to every digestion batch randomly during the whole measurement. A high sample recovery between 95 and 105% was reported and meet the requirement of sample recovery between 90 and 110% in Quality Assurance/Quality Control (QA/QC) compliance of DZ/T 0130-2006 [9].

### 2.3. Assessment Method

The concentration of metals in fish is affected by a variety of factors, of which fish length is an important factor [11]. Moreover, the length of fish is often used as an intuitive reference for angling activities, and also a common reference for the consumers to buy fish. Hence, in this study, 10–35 cm length of fish was divided into five length ranges. Fish with a length less than 10 cm were grouped, as the muscle tissues of small fish were mixed for the determination of metals. Most of the WFAs in the watersheds of Liuzhou city are less than 35 cm in length, which lead to only five fish over 35 cm in length in 171 samples. Based on this, the last length range was set to 35–51 cm.

#### 2.3.1. Assessment of Metal Pollution

The metal pollution index (Pi) is applied to assess the contamination of metals among the different lengths of WFAs [9,19,20]. The Pi of Cd, Cr, Cu, Pb, Zn, As and Se is calculated by the following Equation (1):

Pi = Ci/Csi(1)

where, Pi is the monomial pollution index of metal i; Ci is the content of metal i in the fish samples (mg/kg of wet weight); Csi is the threshold value of metal i in the fish samples. The Csi of Cd, Cr and Pb refer to the mandate of National Food safety Standard-Maximum Residue Limits of Contaminants in Food of China [21]. The Csi of Cu, Hg (total) and As (total) follow the limits of Safety Qualification for Agricultural Product-Safety Requirements for Non-environmental Pollution Aquatic Products of China [22].

The Pi value is divided into four pollution levels [9,19,20,23]. Pi < 0.2 suggests no significant pollution, 0.2 < pi < 0.6 indicates minor pollution, 0.6 < pi < 1 represents a moderate pollution, and pi > 1 illustrates a severe pollution. The total metal pollution index (MPI) is applied to evaluate the comprehensive pollution status of metals in the fish samples. The concentration of metal “n” is represented by Cn, and MPI is calculated by Equation (2):

MPI = (C1 × C2 × C3 ×……Cn)^1/n^ (2)

#### 2.3.2. Health Risk Assessment of Fish Consumption

The target hazard quotient (THQ) is applied to evaluate the health risk of metals that exceed the limits established by relative legislation [24]. THQ is the ratio between the exposure contaminant and the reference dose (RfD):

(3) $$\mathrm{THQ}=\frac{\mathrm{EF}\times \mathrm{ED}\times \mathrm{IRd}\times C}{\mathrm{RfD}\times \mathrm{BW}\times \mathrm{AT}}$$

The calculation parameters of children and adults involved in Equation (3) are presented in Table 2. When the THQ of Hg was calculated, the total concentration of Hg in the muscle tissue was assumed to be equal to that of MeHg, as employed in previous studies [9,25]. The total target hazard quotient (TTHQ) equals the sum of the THQ value of each metal, which can be represented the following Equation (4):

TTHQ = ∑THQ(4)

The exposed population is unlikely to suffer apparent adverse effects when the value of THQ is below 1. A THQ value of >1 indicates that the level of exposure is more than the RfD, and effective interventions, and hence it suggests protective measurements [8].

### 2.4. Statistical Analysis

Excel 2010 (Microsoft, Redmond, Washington DC, USA) and SPSS package 22, (SPSS, Chicago, IL, USA) were used for data processing and analysis. The relationship between the properties of fish and metals were considered significant by the Pearson correlation when *p* < 0.05. The tables and figures were carried out using OriginPro 8 (Electronic Arts Inc, Redwood, Washington DC, USA) and Coreldraw X7 (Corel, Ottawa, Canada).

## 3. Results and Discussion

### 3.1. The Concentration of Metals in the Fish Samples

#### 3.1.1. Analysis of Concentration of Metals in the Fish Samples

The basic statistics of target metals in the WFAs and the corresponding limits of metals recommended by China and international organizations are shown in Table 3. Eight metals were detected in all the WFAs, and the concentrations of these metals were found to be significantly different. The mean concentrations of metals in the fish samples decreased in the order of Zn > Cr > Cu > Se > Cd > As > Hg > Pb, where Zn was the most abundant metal (109.294 mg/kg, wet weight). A comparison with the concentration of these metals in fish during recent years from other study areas is summarized in Table 3. Compared with the concentration of metals in fish from other areas in China, the mean concentrations of Zn, Cr, and Cd in WFAs from Liuzhou city were much higher than the reported values in Yellow River Estuary [30]. Also, they were higher than the values observed in some parts of Northeast China, including Liaoning, Jilin, Heilongjiang province, and Inner Mongolia Autonomous Region [18]. However, the average levels of Cu, Pb, Zn, Cr, Cd, As, and Hg in WFAs from Liuzhou city were significantly lower than in Shanghai city [31]. These observations indicate that the concentration of essential element Zn and toxic elements Cr and Cd were generally high, and the levels of toxic elements Pb, As, and Hg were relatively low in the current study. Compared with the concentration of metals in fish from other countries, the concentrations of Cu, Pb, Cr, and Cd in WFAs during this study were significantly lower than in Ganga River, India [32]. The concentration of Se was slightly higher than that in Río de la Plata Estuary, Brazil [33]. However, the concentration of Zn was significantly higher than that in Río de la Plata Estuary, Brazil, and Ganga River, India [32,33]. The pollution levels of Cr and Cd in wild fish were noticeably higher than in River Kabul, Pakistan [34]. The data mentioned above illustrate that the pollution levels of Zn, Cr, and Cd in fish obtained from these study areas were relatively serious by comparing with a few other countries. This reflects the characteristics of metals pollution connected with the sewage discharge in Liuzhou city and some mining activities in the upstream of Liujiang River and also confirmed relatively severe pollution of some metals in WFAs. The concentration of Se in the fish samples was slightly high, which could be correlated to the difference in the geochemical background of Se in different countries. According to the Maximum Residue Limit (MRL) of metals in fish by China (General Administration of Quality Supervision and China Food and Drug Administration) and other international organizations (ATSDR, FAO/WHO, FDA, and EU) [35,36,37,38,39], though the mean concentrations of eight metals in the samples were relatively low, the concentrations of Zn, Cr, and Cd in WFAs were beyond the MRL. The maximum concentrations of Cr (4.226 mg/kg, wet weight) and Cd (0.196 mg/kg, wet weight) in WFAs were close to twice as that of MRL, as recommended by China. Whereas, the maximum concentrations of Zn (109.294 mg/kg, wet weight) and Cd (0.196 mg/kg, wet weight) in the samples were over twofold and close to fourfold of MRL, respectively, as recommended in Europe.

Zn is an essential microelement to maintain the normal physiological functions of the human body [40], but its excessive accumulation could cause few toxicological diseases [41], such as increasing the potential risks of gastrointestinal toxicity [42]. In general, centralized industrial production commonly leads to the changes in the chemical composition of nearby ecosystems [43], such as zinc mining, natural ores, galvanizing plants, machine manufacturing, and municipal wastewater treatment plants, attributed to increasing the concentration of Zn in the aquatic environment [42,44,45]. The relatively high concentration of Zn among WFAs was inevitably affected by the mining activities taking place in the upstream of the Liujiang River, and a large amount of Zn-containing sewage was discharged from the industrial production in the Liuzhou city. Concerning the high concentration of toxic metal Cr in WFAs, it is an important and indispensable metal element for the production of steel parts and electroplating [46]. Steel production and automobile manufacturing are the major industries of Liuzhou city, which discharge a large amount of sewage with high concentration of Cr. Therefore, a higher emission of Cr can be accounted for the high concentration of Cr in WFAs from the study area [9]. Similarly, the high level of Cd in WFAs was affected by the upstream polluted waters and industries in Liuzhou. Longjiang River, as the upper reach of Liujiang River, is enriched with various polymetallic mineral resources, where numerous large-scale mining and processing activities are continued for many years, leading to the higher levels of various metals in Longjiang River [16,17,47], which led to Cd pollution during 2012 in Longjiang River. Hence, the accumulation of various metals in wild fish was increased to some extent by human activities in the upstream of the Liujiang River.

#### 3.1.2. Correlation Between the Length of Fish and Concentration of Metals

The correlation between the characteristics of fish and concentration of metals in WFAs is shown in Table 4. A significant correlation could be noted between almost all metals, suggesting possible similar sources of these metals in fish [48]. This result is consistent with the pollution of the Liujiang River, which is attributed to the mining activities and industrial production of Liuzhou to a greater extent [16,17,42]. A clear correlation of fish length was found with Cu, Zn, and Cr, and a significant correlation of fish weight was found with Cu, Zn, and Cd. The above outcome is suggesting the bioenrichment of these metals among fish is in line with the effect of biological dilution. The fish length and weight have no significant correlation with the concentrations of As, Hg, and Se in fish, implying that the enrichment of these metals is relatively constant in the whole lifespan of angling fish.

### 3.2. Concentration of Metals in fish with Different Length Ranges

**Figure 2:**
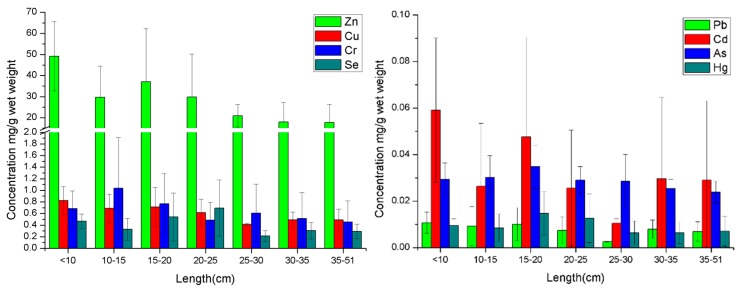
The concentration of metals in different length ranges of fish.

The concentrations of metals in fish with different length ranges determined during this study are shown in Figure 2. The levels of metals in the collected fish with different length ranges are different, and the concentration of metals decreased with the growth of wild fish. The maximum concentrations of Zn, Cu, and Cd were found to be 49.197, 0.821, and 0.059 mg/kg, respectively, in the length range of <10 cm, and the concentration of Cr was the highest (1.038 mg/kg) in the length range of 10–15 cm compared to other length ranges. Hence, the concentrations of these metals in adult wild fish were lower than that in juvenile wild fish, indicating an obvious biological dilution obtained with Cu, Zn, Cr, and Cd in WFAs during the growth process. A similar characteristic of the distribution of both Hg and As in WFAs was observed, and the maximum concentrations of which were 0.015 and 0.035 mg/kg respectively in the length range of 15–20 cm. The biological dilution of metals in fish is affected by various external factors, such as living and feeding habits [30], and also by internal factors such as the rate of growth and metabolism. In this study, a higher concentration of toxic elements Cr, Cd, Hg, and As was noted in these fish with the length range of 10–20 cm. On the one hand, the growth speed of fish with the length range of 10–20 cm is slower compared to the length range of lower than 10 cm; on the other hand, the fat storage in fish with the length range of 10–20 cm is lower than that of beyond 20 cm. Therefore, the metabolic rate of these toxic metals is commonly low in fish with the length range of 10–20 cm, which led to a higher concentration of most of the toxic metals in these WFAs. However, the biological dilution of essential elements, Zn and Cu, were slightly different from these toxic elements in wild fish. Since Zn and Cu play a critical role in the growth of fish and development of organs [7], a large number of essential elements, in particular Zn, are enriched in the fast-growing juvenile fish [49]. In addition, the juvenile fish below the length range of 10 cm usually display relatively weak dilution effects for metals, which could be attributed to the low metabolic rate of metals due to the underdeveloped organs. Hence, the concentrations of Zn and Cu were the highest in fish samples with the length range of <10 cm. It is known from earlier investigations that when humans are exposed to these toxic elements for a long time could lead to diseases or even death [42]. This shows that generally, higher concentrations of these toxic elements were found in fish with the length range of 10–20 cm, guiding the consumers of wild fish in avoiding toxic metals poisoning to some extent.

### 3.3. Degree of Metals Pollution in Fish with Different Length Ranges

**Figure 3:**
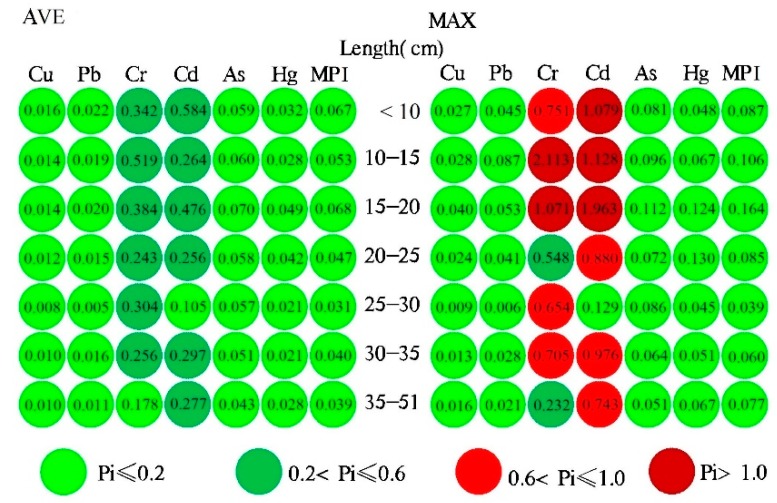
Pollution index (Pi) and total metal pollution index (MPI) in fish with different length ranges.

Pi is an index used to evaluate the pollution levels of various aquatic products and Figure 3 exhibits the levels of Pi in fish with different length ranges. The Pi levels of eight metals followed the order: Cr > Cd > As > Hg > Pb > Cu, and only the Pi of Cr and Cd displayed significantly higher than 0.2, illustrating that pollution in WFAs by Cr and Cd is common. According to the average Pi values, the pollution levels of Cr and Cd in WFAs with the length range of <20 cm were higher compared to the length range of >20 cm, which agrees with the previous analysis. The pollution of Cr in the collected fish with the length range of <35 cm was at moderate levels. Except for fish with the length range of 25–30 cm, slight pollution of Cd was found in the fish for the remaining length ranges. Despite the average values of Pi for Cr and Cd were below 0.2 in some samples, it does not mean that there is slight contamination of these metals in WFAs and hence should not be ignored. However, contamination with Cu, Pb, As, and Hg was not observed in most of the fish samples with different length ranges as the mean Pi values of these metals were much less than 0.2. According to the maximum Pi values, the pollution of Cr and Cd in some of the wild fish could be more severe with the maximum value of Pi > 1.0. A part of fish samples with the length below 20 cm was with the moderate to severe contamination, where the maximum Pi values of Cr and Cd were up to 2.113 and 1.963, respectively, which were higher than the Pi values in other length ranges. The pollution levels of Cr and Cd in different length ranges of the obtained fish were in the order of 10–15 cm > 15–20 cm > 10 cm > 30–35 cm > 20–25 cm > 35–51 cm > 25–30 cm, inferring the pollution degree of metals in WFAs with different length ranges. It is particularly notable that the severe pollution of Cr and Cd were found in fish samples with a length range of 10–20 cm. However, since the intake of Se greatly impairs the intake of Cr [9], and the significantly negative correlation between Se and Cr (Table 4), the wild fish with the length range of 20–25 cm were less influenced with the pollution of Cr compared to other length ranges. Only the WFAs with the length range of 25–30 cm were not contaminated by Cd due to its maximum value of Pi (0.129). The maximum Pi values of Cu, Pb, As, and Hg were below 0.2, indicating that WFAs were hardly contaminated by these metals. Based on the maximum MPI values of lower than 0.2, the contamination of total metals in WFAs has not been noted. Since a small part of wild fish with the length range of 10–20 cm in the study areas were close to slight pollution, and hence a vigilance on the pollution of WFAs with the length range of 10–20 cm still should be continued. Overall, the obtained results revealed that WFAs from Liujiang River and its tributary the Luoqingjiang River were widely polluted to varying levels by Cr and Cd, and severe pollution was more likely to be found in fish with the length range lower than 20 cm, in particular 10–20 cm.

According to an earlier study [9], more than half of the WFAs collected from five sampling points were contaminated by Cd and Cr to varying degrees. The wild fish with the length range of 10–20 cm accounted for 49.1 % of the total fish samples distributed in all the collection sites. In addition, the fish samples included 14 species, of which 11 species (including *Pseudohemiculter dispar*, *Cyprinus carpio*, and *Ervthroculter hypselonotus*) suffered from different levels of Cd and Cr pollution. Hence, although the concentration of metals in wild fish is affected by the differences in the sampling points and fish species, wild fish with length ranges of 10–20 cm was more likely to be contaminated by Cd and Cr. 

### 3.4. Health Risk Assessment of Metals in Fish with Different Length Ranges

**Figure 4:**
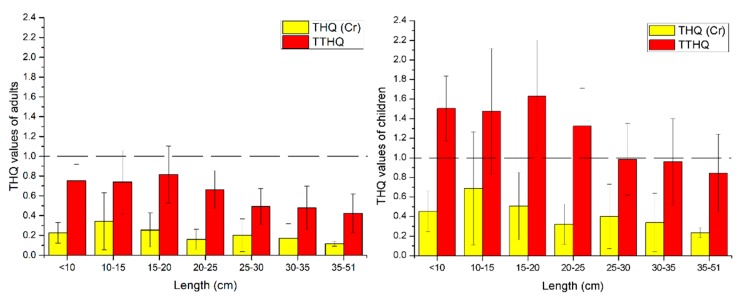
Target hazard quotient (THQ) of Cr and total target hazard quotient (TTHQ) in fish with different length ranges.

The THQ values of eight metals from the consumption of wild fish were calculated based on adults and children, as shown in Table 5. It could be observed that the THQ values of metals decreased in the order of Cr > Zn > Hg > As > Se > Cd > Cu > Pb. The average THQ values of all the metals were below 1, indicating that the health risks of consuming wild fish might not be high in terms of individual metal. However, the maximum THQ value of Cr over 1 in some fish may cause concern. Although previous analysis has demonstrated that most wild fish were polluted by Cd [9], all the THQ values of Cd were below 1, and the relatively high value of Oral Reference Dose for Cd is the main reason for a decrease in the THQ values of Cd. The maximum THQ values of Zn, Hg, As, Se, Cd, Cu, and Pb were less than 1, suggesting that these metals from wild fish hardly pose potential health risks to adults and children. Hence, the THQ of Cr contributes most to TTHQ. The THQ of Cr and TTHQ for adults and children have been displayed in Figure 4. The health risks related to different length ranges of WFAs were in the order: 15–20 cm > 10 cm > 10–15 cm > 20–25 cm > 25–30 cm > 30–35 cm > 35–51 cm, suggesting that the relatively high health risks should be attributed to eating small fish, of which with the length range of 15–20 cm were identified as the most severe health risk. For adults, even though the maximum values of THQ of Cr and TTHQ appeared to be more than 1 with the length ranges of 10–15 cm and 10–25 cm, respectively, the mean values of THQ and TTHQ of Cr were less than 1. Hence, these results indicated that it could be generally safe for adults to eat all length ranges of WFAs, but the intake of small fish should be controlled. However, the levels of health risks for children are much higher than in adults. Although the THQ values of Cr for children were less than 1 with all length ranges, the TTHQ values were over 1 with the length ranges of <10 cm, 10–15 cm, 15–20 cm, and 20–25 cm. This suggests that for children the consumption of wild fish in these length ranges could cause higher health risks. Meanwhile, the intake of other length ranges of WFAs should also be strictly controlled.

**Figure 5:**
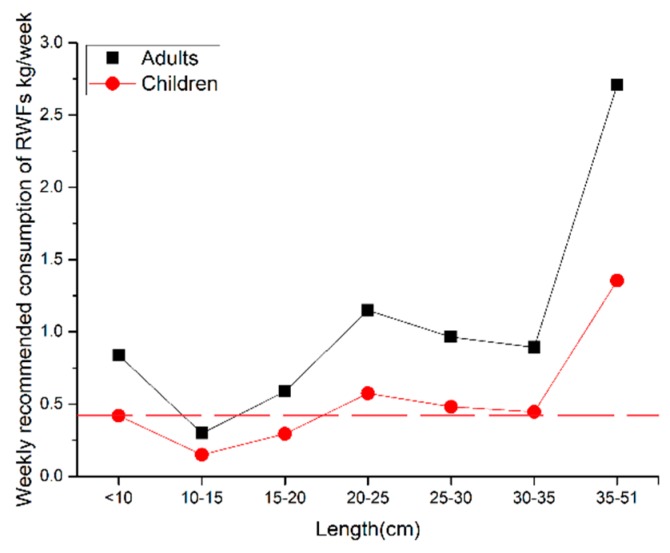
Weekly recommended consumption of fish (kg/week) with different length ranges.

According to a previous report, the amount of fish consumption among urban residents in Guangxi province is 0.42 kg/week [26]. However, depending on the consumption, the residents are likely to be exposed to various levels of health risks. In order to accurately advise on the consumption of WFAs, the maximum concentration in Equation (3) was adopted to the concentration of the metals, in which other parameters were unchanged for calculating the maximum allowance of wild fish consumption per week [9]. From the earlier analysis, it has been noted that the Cr contamination could be considered as the most harmful to the consumption of fish, as the THQ of Cr contributes most to the TTHQ (Table 5). Therefore, the maximum concentration of Cr was used to calculate the weekly maximum allowance of fish consumption. As shown in Figure 5, the weekly maximum allowance of fish consumption with different length ranges were in the order of 35–51 cm > 20–25 cm > 25–30 cm > 30–35 cm > 10 cm > 15–20 cm > 10–15 cm. For adults, the weekly maximum allowance of fish consumption with the length range of 15–20 cm was slightly lower than 0.42 kg/week, which indicated the necessity of vigilance on the consumption of wild fish with this length range. However, the consumption of fish with the remaining length ranges was recognized as safe for adults. For children, the weekly maximum allowance of fish consumption with the length range of <20 cm was significantly lower than 0.42 kg/week, suggesting the necessity for caution in the consumption of fish. Also, the weekly maximum allowance of fish consumption with the length range of 20–51 cm was slightly higher than 0.42 kg/week, indicating safer fish consumption for children. Based on these results, the maximum safe intake of WFAs with the length ranges of < 10 cm, 10–15 cm, and 15–20 cm should be controlled below 0.419 kg/week, 0.149 kg/week, and 0.294 kg/week, respectively, for children. Meanwhile, the maximum safe intake of WFAs with the length range of 10–15 cm should be controlled below 0.298 kg/week for adults. Overall, the WFAs with the length range of <20 cm were less safe to eat and should be avoided. The WFAs with the length range of 20–51 cm were relatively safe to eat, especially for children. Furthermore, as the weekly recommended consumption of wild fish with the length range of 15–20 cm was lower than the current rate of fish consumption, the targets of angling activities should be evaded for wild fish with these length ranges in the waterways of Liuzhou city.

## 4. Conclusions

The findings from this study provide useful information for the health risk assessment relating to the consumption of WFAs, which is attributed to the presence of elevated concentrations of metals in the waterways of Liuzhou city (China). Fish samples (171) collected from the Liujiang River and its tributary the Luoqingjiang River had relatively high concentrations of Zn, Cr, and Cd of up to 109.294 mg/kg, 4.226 mg/kg, and 0.196 mg/kg (wet weight), respectively, and were observed to exceed MRL. The Pi analysis results indicated that WFAs with different length ranges, particularly with the length range of 10–20 cm (maximum value of Pi > 1.0), were commonly polluted to different degrees only by Cr and Cd. The THQ of Cr contributed the most to the TTHQ. For adults, most of the THQ values were lower than 1, suggesting that there is no obvious health risk for the consumption of wild fish. However, the intake of WFAs should be controlled. Nevertheless, for children, values of TTHQ above 1 were distributed in most of the length ranges, especially for 10–15 cm (1.627), indicating that the consumption of wild fish could cause significantly high health risks due to the consumption of WFAs. The weekly recommended consumption results indicated the detailed, safe consumption of wild fish with different ranges for adults and children. The weekly recommended consumption of WFAs with the length ranges of 10–15 cm for adults and children was only 0.298 kg/week and 0.149 kg/week, respectively, which were significantly lower than the current rate of fish consumption (0.42 kg/week). Given the relatively low consumption of fish with the length range of 10–15 cm, the angling activities should be restricted to collecting wild fish with the length range of 10–15 cm, and the consumption of wild fish in the 10–15 cm length range should be avoided.

## Figures and Tables

**Table 1:** Details about the fish collected from the Liujiang River and its tributary the Luoqingjiang River.

Species	Station	Length (cm)		Weight (g)		Number	Feeding habit	Habitat
		Min	Max	Min	Max			
*Pseudohemiculter dispar*	S1, S2, S4 and S5	5	37	20.6	105.9	106	Omnivore	Pelagic
*Cyprinus carpio*	S2, S3 and S5	8	41	26.3	887.0	21	Omnivore	Demersal
*Ervthroculter hypselonotus*	S1, S2 and S3	6	34	38.7	79.2	19	Carnivore	Pelagic
Others	S2, S3 and S5	8.5	51	19.3	1705.2	25	Omnivore, Carnivore and Herbivore	Pelagic, Demersal and Shallow area

**Table 2:** Definition and reference values for some of the parameters used in the health risk assessment of metals.

Parameter		Children	Adult	Reference
Ingestion Rate (IRd, g/day)		59.6	59.6	[26]
Exposure Frequency (EF, days/year)		365	365	[18,27]
Exposure Duration (ED, year)		6	70	[18,27]
Body Weigh (BW, Kg) ^1^		30	60	[18,28]
Average Time (AT. day/year)		6 × 365	70 × 365	[18,27]
Oral Reference Dose (RfD, μg/kg/day)	Cu	40	40	[25,29]
	Pb	4		
	Zn	300	300	
	Cr	3	3	
	Cd	1	1	
	As ^2^	0.3	0.3	
	Hg	0.1	0.1	
	Se	5	5	

^1^ China people’s average body weight during the study; ^2^ The RfD value does belong to MeHg.

**Table 3:** The concentration of metals in WFAs and the corresponding maximum residue limits (mg/kg of wet weight).

Region		Cu	Pb	Zn	Cr	Cd	As	Hg	Se
Current study		0.245–2.002	0.0041–0.044	7.532–109.294	0.040–4.226	0.001–0.196	0.010–0.056	0.002–0.039	0.052–1.971
		0.660	0.009	31.649	0.796	0.034	0.030	0.010	0.422
Yellow River Estuary, China [30]		0.15–2.88	0.04–0.79	7.06–29.8	0.02–2.4	0.001–0.09	-	-	-
Northeast China [18]		1.8	0.062	12.6	0.35	0.0047	0.17	0.13	-
Shanghai, China [31]		0.15–12.3	nd–1.43	4.3–85.7	0.44–2.68	nd–0.31	nd–0.47	0.02–0.42	-
		2.95	0.35	34.3	1.23	0.06	0.18	0.15	-
Ganga River, India [32]		1.12–2.14	0.34–2.03	9.43–22.38	2.41–4.90	0.22–0.71	-	-	-
Río de la Plata Estuary, Brazil [33]		-	-	17.6	-	-	14.5	-	0.4
River Kabul, Pakistan [34]		-	0.476	-	0.29	0.028	-	-	-
MRL	China	50	0.5	NA	2.0	0.1	0.5	0.3	NA
	International	30	0.5	50	8.0	0.05	1.0	0.5	2.0

Note: nd represent below detection limit; NA represent unreferenced standard; Chinese MRL includes GB2762-2017 and GB18406.4-2001; Other MRLs include ASSDR, FAO/WHA, FDA, and EU.

**Table 4:** Correlation matrix between the characteristics of fish and concentration of metals in fish.

Factor	Length (cm)	Weight (g)	Cu	Pb	Zn	Cr	Cd	As	Hg	Se
Length (cm)	1.000	0.718 **	−0.347 **	−0.196	−0.377 **	−0.248 *	−0.202	−0.208	−0.098	−0.051
Weight (g)	-	1.000	−0.263 *	−0.156	−0.239 *	−0.225	−0.242 *	−0.216	−0.197	−0.083
Cu	-	-	1.000	0.201	0.524 **	0.237 *	0.380 **	0.317 **	−0.032	0.092
Pb	-	-	-	1.000	0.111	0.310 **	0.127	0.034	0.195	−0.155
Zn	-	-	-	-	1.000	0.062	0.515 **	0.050	0.178	0.251 *
Cr	-	-	-	-	-	1.000	−0.109	0.125	−0.008	−0.295 *
Cd	-	-	-	-	-	-	1.000	0.038	0.419 **	0.345 **
As	-	-	-	-	-	-	-	1.000	−0.115	0.206
Hg	-	-	-	-	-	-	-	-	1.000	0.249 *
Se	-	-	-	-	-	-	-	-	-	1.000

** Correlation is significant at 0.01 level (2-tailed); * Correlation is significant at 0.05 level (2-tailed).

**Table 5:** Ranges and means of target hazard quotients (THQ) of metals in fish with different length ranges.

Length (cm)	Cu	Pb	Zn	Cr	Cd	As	Hg	Se	TTHQ
	**Adults**								
<10	0.006–0.033	0.0010–0.0056	0.053–0.207	0.098–0.498	0.007–0.107	0.033–0.135	0.026–0.142	0.040–0.115	0.309–0.909
	0.202	0.0027	0.161	0.226	0.058	0.098	0.094	0.091	0.752
10–15	0.006–0.034	0.0003–0.0109	0.032–0.234	0.021–1.400	0.002–0.112	0.044–0.159	0.019–0.201	0.010–0.152	0.269–1.688
	0.017	0.0023	0.098	0.344	0.026	0.100	0.085	0.066	0.738
15–20	0.010–0.497	0.0002–0.0066	0.032–0.362	0.013–0.709	0.012–0.195	0.068–0.185	0.040–0.368	0.048–0.391	0.362–1.614
	0.018	0.0025	0.123	0.254	0.047	0.115	0.147	0.107	0.814
20–25	0.011–0.294	0.0002–0.0050	0.034–0.245	0.055–0.363	0.006–0.087	0.059–0.120	0.022–0.389	0.031–0.373	0.387–1.008
	0.015	0.0018	0.099	0.161	0.025	0.096	0.126	0.138	0.662
25–30	0.010–0.011	0.0006–0.0007	0.044–0.830	0.066–0.433	0.008–0.013	0.048–0.142	0.028–0.135	0.022–0.067	0.244–0.680
	0.010	0.0006	0.069	0.201	0.010	0.095	0.064	0.042	0.492
30–35	0.008–0.016	0.0009–0.0035	0.028–0.114	0.079–0.467	0.002–0.097	0.066–0.105	0.024–0.153	0.034–0.111	0.286–0.852
	0.012	0.0020	0.060	0.170	0.029	0.084	0.063	0.060	0.480
35–51	0.006–0.204	0.0004–0.0026	0.029–0.087	0.099–0.154	0.001–0.074	0.050–0.085	0.020–0.199	0.039–0.078	0.251–0.670
	0.012	0.0013	0.057	0.118	0.027	0.071	0.082	0.054	0.422
	**Children**								
<10	0.012–0.066	0.0019–0.4133	0.106–0.413	0.196–0.995	0.014–0.214	0.066–0.270	0.053–0.285	0.081–0.231	0.618–1.817
	0.040	0.0054	0.322	0.453	0.116	0.195	0.189	0.183	1.503
10–15	0.012–0.069	0.0006–0.0217	0.065–0.468	0.042–2.799	0.003–0.224	0.089–0.319	0.039–0.402	0.021–0.304	0.537–3.376
	0.034	0.0047	0.196	0.688	0.052	0.200	0.170	0.131	1.476
15–20	0.021–0.099	0.0004–0.0131	0.065–0.724	0.027–1.418	0.024–0.390	0.137–0.371	0.079–0.737	0.096–0.783	0.724–3.229
	0.035	0.0050	0.245	0.509	0.095	0.231	0.294	0.214	1.627
20–25	0.022–0.059	0.0005–0.0101	0.068–0.490	0.111–0.726	0.011–0.175	0.118–0.239	0.044–0.777	0.062–0.747	0.774–2.017
	0.031	0.0037	0.197	0.321	0.051	0.192	0.252	0.276	1.324
25–30	0.019–0.022	0.0012–0.0014	0.088–0.166	0.133–0.866	0.017–0.026	0.096–0.284	0.055–0.270	0.044–0.133	0.489–1.360
	0.020	0.0013	0.139	0.402	0.021	0.189	0.127	0.084	0.984
30–35	0.015–0.033	0.0017–0.0071	0.057–0.229	0.157–0.934	0.004–0.194	0.132–0.211	0.047–0.305	0.068–0.222	0.572–1.705
	0.024	0.0040	0.119	0.339	0.059	0.168	0.126	0.120	0.960
35–51	0.012–0.041	0.0007–0.0053	0.058–0.174	0.198–0.308	0.003–0.148	0.101–0.170	0.041–0.398	0.078–0.156	0.501–1.399
	0.025	0.0027	0.113	0.235	0.055	0.142	0.165	0.107	0.844

## References

[1] Sinha A.K., Kumar V., Makkar H.P.S., De Boeck G., Becker K. (2011). Non-starch polysaccharides and their role in fish nutrition—a review. Food Chem..

[2] Gu Y.G., Lin Q., Wang X.H., Du F.Y., Yu Z.L., Huang H.H. (2015). Heavy metal concentrations in wild fishes captured from the South China sea and associated health risks. Mar. Pollut. Bull..

[3] Food and Agriculture Organization of the United Nations (FAO) (2008). The State of World Fisheries and Aquaculture.

[4] Lv D.Z., Tang Y.J., Yuan H.Q., Li J.G. (2013). Research on pollution status and risk assessment of environmental hormone in aquaculture seawater of Shenzhen, China. Adv. Mater. Res..

[5] Liu X., Steele J.C., Meng X.Z. (2017). Usage, residue, and human health risk of antibiotics in Chinese aquaculture: A review. Environ. Pollut..

[6] Liu J.L., Xu X.R., Ding Z.H., Peng J.X., Jin M.H., Wang Y.S., Hong Y.G., Yue W.Z. (2015). Heavy metals in wild marine fish from South China Sea: Levels, tissue- and species-specific accumulation and potential risk to humans. Ecotoxicology.

[7] Varol M., Sünbül M.R. (2018). Multiple approaches to assess human health risks from carcinogenic and non-carcinogenic metals via consumption of five fish species from a large reservoir in Turkey. Sci. Total Environ..

[8] Zhong W., Zhang Y., Wu Z., Yang R., Chen X., Yang J., Zhu L. (2018). Health risk assessment of heavy metals in freshwater fish in the central and eastern North China. Ecotoxicol. Environ. Saf..

[9] Miao X.Y., Hao Y.P., Tang X., Xie Z.Q., Liu L.P., Luo S.W., Huang Q.B., Zou S.Z., Zhang C.L., Li J. (2020). Analysis and health risk assessment of toxic and essential elements of the wild fish caught by anglers in Liuzhou as a large industrial city of China. Chemosphere.

[10] Alkan N., Muammer A., Gedik K. (2012). Comparison of metal accumulation in fish species from the southeastern black sea. Bull. Environ. Contam. Toxicol..

[11] Sackett D., Cope W., James R., Aday D. (2013). The influence of fish length on tissue mercury dynamics: Implications for natural resource management and human health risk. Int. J. Environ. Res. Public Health.

[12] Affandi F.A., Ishak M.Y. (2019). Impacts of suspended sediment and metal pollution from mining activities on riverine fish population—a review. Environ. Sci. Pollut. Res..

[13] Canli M., Atli G. (2003). The relationships between heavy metal (Cd, Cr, Cu, Fe, Pb, Zn) levels and the size of six Mediterranean fish species. Environ. Pollut..

[14] Mortazavi M.S., Sharifian S. (2012). Metal Concentrations in Two Commercial Fish from Persian Gulf, in Relation to Body Length and Sex. Bull. Environ. Contam. Toxicol..

[15] Miao Y., Kong X., Li C. (2018). Distribution, sources, and toxicity assessment of polycyclic aromatic hydrocarbons in surface soils of a heavy industrial city Liuzhou. China. Environ. Monit. Assess..

[16] Zhang Q.H., Wei Y.Z., Cao J.H., Yu S. (2018). Heavy metal pollution of the drinking water sources in the Liujiang River Basin, and related health risk assessments. Environ. Sci..

[17] Lan X.L., Ning Z.P., Xiao Q.X., Huang Z.Y., Liu Y.Z., Xiao T.F., Zhao Y.L., Shi Liang W.U. (2018). Spatial Distribution, Sources and bioavailability of heavy metals in the surface sediments of Longjiang River, Southern China. Environ. Sci..

[18] Fu L., Lu X., Niu K., Tan J., Chen J. (2019). Bioaccumulation and human health implications of essential and toxic metals in freshwater products of Northeast China. Sci. Total Environ..

[19] Liu Y., Fu Q., Gao J., Xu W.G., Qin W.H. (2013). Concentrations and safety evaluation of heavy metals in aquatic products of Yancheng, Jiangsu province. Environ. Sci..

[20] Zhu F., Qu L., Fan W., Wang A., Hao H., Li X., Yao S. (2015). Study on heavy metal levels and its health risk assessment in some edible fishes from Nansi Lake, China. Environ. Monit. Assess..

[21] China Food and Drug Administration (2012). National and Food Safety Standard-Maximum Residue Limits of Contaminants in Food (GB 2762-2017).

[22] General Administration of Quality Supervision (2001). Safety Qualification for Agricultural Product-Safety Requirements for Non-environmental Pollution Aquatic Products (GB 18406.4-2001).

[23] Yap C.K., Ismail A., Tan S.G. (2003). Background concentrations of Cd, Cu, Pb and Zn in the green-lipped mussel Perna viridis (Linnaeus) from Peninsular Malaysia. Mar. Pollut. Bull..

[24] United States Environmental Protection Agency (EPA) (2000). U.S. Risk-Based Concentration Table.

[25] Jiang Z., Xu N., Liu B., Zhou L., Wang J., Wang C., Dai B., Xiong W. (2018). Metal concentrations and risk assessment in water, sediment and economic fish species with various habitat preferences and trophic guilds from Lake Caizi, Southeast China. Ecotoxicol. Environ. Saf..

[26] Chen X., Tang Z., Han Y. (2005). Investigation on Dietary Intake and Nutritional Status of Residents in Guangxi Province. China Public Health.

[27] U.S. EPA (2000). Guidance for Assessing Chemical Contaminant Data for Use in Fish Advisories.

[28] Wang Z., Duan X., Liu P., Nie J., Zhang J. (2009). Human exposure factors of Chinese people in environmental health risk assessment. Res. J. Environ. Sci..

[29] Agusa T., Kunito T., Sudaryanto A., Monirith I., Kan-Atireklap S., Iwata H., Ismail A., Sanguansin J., Muchtar M., Tana T.S. (2007). Exposure assessment for trace elements from consumption of marine fish in Southeast Asia. Environ. Pollut..

[30] Liu H., Liu G., Wang S., Zhou C., Yuan Z., Da C. (2018). Distribution of heavy metals, stable isotope ratios (δ^13^C and δ^15^N) and risk assessment of fish from the Yellow River Estuary, China. Chemosphere.

[31] Zhang F.F., Yang S.Q., Xu Y.P., Zhou Z., Huang Q.H. (2017). Contamination of heavy metals in game fishes in Shanghai and fish consumption safety assessment. China Environ. Sci..

[32] Siddiqui E., Verma K., Pandey U., Pandey J. (2019). Metal contamination in seven tributaries of the Ganga River and assessment of human health risk from fish consumption. Arch. Environ. Contam. Toxicol..

[33] Avigliano E., Maichak de Carvalho B., Invernizzi R., Olmedo M., Jasan R., Volpedo A.V. (2019). Arsenic, selenium, and metals in a commercial and vulnerable fish from Southwestern Atlantic estuaries: Distribution in water and tissues and public health risk assessment. Environ. Sci. Pollut. Res. Int..

[34] Ali H., Khan E. (2018). Assessment of potentially toxic heavy metals and health risk in water, sediments, and different fish species of River Kabul, Pakistan. Hum. Ecol. Risk Assess..

[35] Hosseini S.M., Sobhanardakani S., Navaei M.B., Kariminasab M., Aghilinejad S.M., Regenstein J.M. (2013). Metal content in caviar of wild Persian sturgeon from the southern Caspian Sea. Environ. Sci. Pollut. Res..

[36] Hosseini S.V., Sobhanardakani S., Tahergorabi R., Delfieh P. (2013). Selected heavy metals analysis of Persian sturgeon’s ( *Acipenser persicus* ) caviar from southern caspian sea. Biol. Trace Elem. Res..

[37] Sobhanardakani S. (2017). Potential health risk assessment of heavy metals via consumption of caviar of Persian sturgeon. Mar. Pollut. Bull..

[38] Sobhanardakani S. (2017). Tuna fish and common kilka: Health risk assessment of metal pollution through consumption of canned fish in Iran. J. Consum. Protect. Food Saf..

[39] Sobhanardakani S., Tayebi L., Hosseini S.V. (2018). Health risk assessment of arsenic and heavy metals (Cd, Cu, Co, Pb, and Sn) through consumption of caviar of *Acipenser persicus* from Southern Caspian Sea. Environ. Sci. Pollut. Res. Int..

[40] Morshdy A.E.M.A., Darwish W.S., Daoud J.R.M., Sebak M.A.M. (2019). Estimation of metal residues in oreochromis niloticus and mugil cephalus intended for human consumption in Egypt: A health risk assessment study with some reduction trials. J. Verbrauch. Lebensm..

[41] Darwish W.S., Ikenaka Y., Nakayama S., Ishizuka M. (2014). The effect of copper on the mrRNA expression profile of xenobiotic-metabolizing enzymes in cultured rat H4-II-E cells. Biol. Trace Elem. Res..

[42] Hajahmadi Z., Younesi H., Bahramifar N., Khakpour H., Pirzadeh K. (2015). Multicomponent isotherm for biosorption of Zn(II), CO(II) and Cd(II) from ternary mixture onto pretreated dried *Aspergillus niger* biomass. Water Resour. Ind..

[43] Niri A.S., Sharifian S., Ahmadi R. (2015). Assessment of Metal Accumulation in Two Fish Species (*Tenualosa ilisha* and *Otolithes ruber*), Captured from the North of Persian Gulf. Bull. Environ. Contam. Toxicol..

[44] Iribar V., Izco F., Tames P., Antigüedad I., Silva A.D. (2000). Water contamination and remedial measures at the Troya abandoned Pb-Zn mine (The Basque Country, Northern Spain). Environ. Geol..

[45] Jones A., Rogerson M., Greenway G., Potter H.A., Mayes W.M. (2013). Mine water geochemistry and metal flux in a major historic Pb-Zn-F orefield, the Yorkshire Pennines, UK. Environ. Sci. Pollut. Res. Int..

[46] Lin W.J. (2010). Characteristics and control of Cr pollution in electroplating wastewater. Environ. Sci. Technol..

[47] Zhao X.M., Yao L.A., Ma Q.L., Zhou G.J., Wang L., Fang Q.L., Xu Z.C. (2017). Distribution and ecological risk assessment of cadmium in water and sediment in Longjiang River, China: Implication on water quality management after pollution accident. Chemosphere.

[48] Zhou J.M., Jiang Z.C., Xu G.L., Qin X.Q., Huang Q.B., Zhang L.K. (2019). Distribution and health risk assessment of metals in groundwater around iron mine. China Environ. Sci..

[49] Papagiannis I., Kagalou I., Leonardos J., Petridis D., Kalfakakou V. (2004). Copper and zinc in four freshwater fish species from Lake Pamvotis (Greece). Environ. Int..

